# Developing an open science ‘mindset’

**DOI:** 10.1080/21642850.2021.2012474

**Published:** 2021-12-26

**Authors:** Martin S. Hagger

**Affiliations:** a Department of Psychological Sciences and Health Sciences Research Institute, University of California, Merced, CA, USA; b Faculty of Sport and Health Sciences, University of Jyväskylä, Jyväskylä, Finland; c School of Applied Psychology, Griffith University, Mt. Gravatt, Australia

**Keywords:** Open science, research transparency, data sharing, pre-registration, replication crisis

## Abstract

**Background:**

Identification of widespread biases present in reported research findings in many scientific disciplines, including psychology, such as failures to replicate and the likely extensive application of questionable research practices, has raised serious concerns over the reliability and trustworthiness of scientific research. This has led to the development of, and advocacy for, ‘open science’ practices, including data, materials, analysis, and output sharing, pre-registration of study predictions and analysis plans, and increased access to published research findings. Implementation of such practices has been enthusiastic in some quarters, but literacy in, and adoption of, these practices has lagged behind among many researchers in the scientific community.

**Advances:**

In the current article I propose that researchers adopt an open science ‘mindset’, a comprehensive approach to open science predicated on researchers’ operating under the basic assumption that, wherever possible, open science practices will be a central component of all steps of their research projects. The primary, defining feature of the mindset is a commitment to open science principles in all research projects from inception to dissemination. Other features of the mindset include the assumption that all components of research projects (e.g. pre-registered hypotheses, protocols, materials, analysis plans, data, and output) will be accessible broadly; pro-active selection of open fora to disseminate research components and findings; open and transparent dissemination of reports of the research findings in advance of, and after, formal publication; and active promotion of open science practices through education, modeling, and advocacy.

**Conclusion:**

The open science mindset is a ‘farm to fork’ approach to open science aimed at promoting comprehensive quality in application of open science, and widening participation in open science practices so that they become the norm in research in health psychology and behavioral medicine going forward.

## Improving research integrity: the evolution of open science

A number of concerns about the integrity, credibility, and transparency of scientific research, particularly in the field of psychology, have been identified in recent years. These concerns have arisen from high-profile ‘failures’ to replicate key research findings (e.g. Hagger, Chatzisarantis, et al., 2016; Harris, Coburn, Rohrer, & Pashler, 2013; Open Science Collaboration, 2015; Pashler & Wagenmakers, 2012; Ritchie, Wiseman, & French, 2012; Schimmack, 2020) and analyses that indicate the presence of biases likely related to how research is produced and published (e.g. Ioannidis, 2005; Simmons, Nelson, & Simonsohn, 2011). This has led to a ‘crisis of confidence’ in psychology, and in other fields, on the validity of research evidence and the systems that produce and disseminate it, and raised questions on the trustworthiness of scientific research among those who consume published research including scientists themselves, governmental organizations and stakeholders, research funders, journalists, and the casual ‘general public’ reader (Anvari & Lakens, 2018; Baker, 2016; Pashler & Wagenmakers, 2012). These issues have compelled researchers and scientists to investigate the extent of the bias in the extant psychology research, and the extent to which findings can be replicated. Such research plays a pivotal role in identifying of the causes of bias and informing the development of practices aimed at minimizing error and bias, and increasing the transparency and precision in the dissemination of research findings (Jamieson, McNutt, Kiermer, & Sever, 2019; Stanley, Carter, & Doucouliagos, 2018; van Aert, Wicherts, & van Assen, 2019).

Studies have identified numerous biases in psychology research indicative of questionable research practices including ‘data dredging’ or ‘p-hacking’ (Head, Holman, Lanfear, Kahn, & Jennions, 2015), hypothesizing after results are known (‘HARK-ing’; Kerr, 1998; Rubin, 2017; Szucs, 2016), and selective reporting (Simmons et al., 2011). Studies have also identified dissemination practices that may introduce bias such as journal reviewers and editors favoring positive, statistically significant findings (Bialystok, Kroll, Green, MacWhinney, & Craik, 2015; Sterling, Rosenbaum, & Weinkam, 1995), and research incentive systems that equate positive findings with success and career progression (Heise & Pearce, 2020; Higginson & Munafò, 2016; Smaldino, 2016). Many of these questionable practices were identified many years previously, but received limited widespread attention (e.g. Sterling et al., 1995; Vevea & Hedges, 1995). However, the advent of more sophisticated bias detection methods and indicators of bias coupled with the high profile of replication failures within the scientific community and the broader media, has led to a groundswell of support and advocacy for solutions.

In response, numerous organizations, working groups, and advisory committees have been set up to develop recommendations and guidelines that aim to improve replicability, reproducibility, credibility, and transparency in research (e.g. Aczel et al., 2020; Appelbaum et al., 2018; Kidwell et al., 2016; Morey et al., 2016; NIH, 2021a; Nosek et al., 2015; NSF, 2021a; Open Science MOOC, 2018; SIPS, 2021; The FOSTER consortium, 2021a). Collectively, these recommendations and practices that are broadly aimed at improving scientific research integrity have become known as ‘open science’. The high profile and widespread dissemination of the reproducibility ‘crisis’ and biases within science, and the increased scrutiny within scientific organizations, means that it is highly unlikely that these issues will be suppressed as before, and there is genuine momentum to instigate real change in research practices within multiple fields, including psychology. Recommendations promulgated by open science advocate groups and organizations are being embraced and implemented by the publishers and funders of scientific research, by researchers’ employers, and by researchers themselves, representing a self-correction of the research practices that lead to problems with reproducibility and the presence of bias (Aczel et al., 2020; Earp & Trafimow, 2015; Kwasnicka et al., 2020; McDiarmid et al., 2021; Pashler & Harris, 2012; Smaldino & McElreath, 2016).

It must, however, be stressed that progress toward universal uptake of advocated open science practices has been slow, and considerable advocacy and promotion work is still needed. For example, basic awareness of open science practices, and the need for change in the way research is conducted is still relatively sparse (Bueno de la Fuente, 2016; Heise & Pearce, 2020). Knowledge of open science practices is also ‘patchy’ and uptake tends to be dependent on whether research groups have a strong open science advocate in their ranks, or whether their particular research or professional organization, or leading journal, has sufficiently embraced open science practices (Armeni et al., 2021). There is also a substantive training deficit – few undergraduate and graduate degree programs include integral or even optional content on open science practices (Jekel et al., 2020; Strand & Brown, 2019). If there is expectation for norms in the practices and methods by which research is typically produce need to change in order to iron out potential sources of bias, it is essential that the next generation of researchers adopt these practices from the get-go. This will effectively introduce a ‘new norm’ in which researchers are ever-mindful of the need to prevent bias and problems with transparency and replication in their research, and instigate means to do so in their study designs from inception to dissemination. Importantly, in order for these changes to ‘stick’ long-term and for science to adopt open research practices that become the norm, there is need for researchers to have open science at the forefront of everything they do. Open science practices should therefore omnipresent in all stages of the research process from the kernel of an idea to the production of a final research report and recommendations for practice, particularly issues of precision in reporting and transparency.

In the current article I advocate just such an approach, which I have called an open science ‘mindset’. This approach suggests that researchers should consider open science from the very outset of their research, and be prepared to consider the open science issues at play at each research stage – a ‘farm to fork’ approach. Importantly, the ‘mindset’ implies that researchers’ should assume that each stage of their research should be open to scrutiny by anyone with an expressed interest in how their research is conducted, an open door or ‘open lab’ approach. The mindset approach is one that I have honed in recent years, arising from my own research experiences and connection with open science, and how I have adjusted my own thinking and research practices and introduced them to the research trainees for whom I am responsible (undergraduate research assistants, graduate students, postdocs). Next, I outline the key experiences that led me to become aware of, adopt, and advocate for, open science practices, and how these experiences reflected the broader issues of transparency and research integrity being explored at the time, and how they led me to change my thinking and approach to research. Then I introduce the open science ‘mindset’, describe its features, and provide some suggestions on how interested researchers might adopt such a mindset.

## What I wish I had known about open science

I only became aware of open science practices, and made changes to implement them in my own research, relatively recently. But it came at a time when the open science movement to reduce bias and improve research precision and integrity was gaining significant momentum (e.g. Baker, 2016; Nosek et al., 2015). My foray into open science was also somewhat serendipitous, the consequence of concerns about bias and replication in my own research, which occurred, in no small part, as a result of other researchers’ investigations that identified those potential biases and raised my awareness of inherent problems in research methods and dissemination. The major catalyst of my interest in open science was a reanalysis and new analysis that was conducted of a meta-analysis that I conducted with my colleagues in the area of self-control (Carter, Kofler, Forster, & McCullough, 2015; Carter & McCullough, 2014), and subsequent replication we conducted with the goal of resolving the matter (Hagger, Chatzisarantis, et al., 2016). Another important stimulus was my career-long interest in the synthesis of research on the correlates of health behaviors using meta-analysis, a program of research with the goal of uncovering the most salient predictors (e.g. Hagger & Chatzisarantis, 2009, 2016; Hagger, Polet, & Lintunen, 2018; Hamilton, van Dongen, & Hagger, 2020; Protogerou, Johnson, & Hagger, 2018; Zhang et al., 2019). Through this research I slowly came to recognize, like many before me, the importance of research integrity, and the necessity to adopt and advocate for open science practices to ensure credibility and transparency in the research evidence I produced with my collaborators going forward. Next I outline those two experiences and how they turned me toward open science; both provide illustrations of key issues of bias and transparency in research in health psychology, and more broadly, and emphasize the need to adopt an open science ‘mindset’ when conducting research.

### The ego-depletion ‘affair’

The first ‘I wish I had known’ experience that led me to the door of open science was my involvement in testing the ‘ego-depletion’ effect in self-control. Just over ten years ago, my late, great colleague, Nikos Chatzisarantis and I were awarded a research grant to examine the effects of choice on self-control. One of pre-dominant theoretical perspectives on self-control at the time was Roy Baumeister and colleagues’ limited resource or ‘strength’ model. In this elegant and intuitively-appealing model, self-control was conceptualized as a limited resource (Baumeister, Bratslavsky, Muraven, & Tice, 1998; Muraven, Tice, & Baumeister, 1998). Individuals were predicted to have good capacity to perform tasks and actions requiring self-control (e.g. resisting temptations, regulating thoughts and emotions, inhibiting dominant responses, suppressing impulses, breaking habits) for a period of time. However, once their self-control ‘resources’ became depleted, their self-control capacity would be impaired leading to a marked reduction in performance on subsequent self-control tasks or even complete failure, a state referred to as ‘ego-depletion’. We conducted some ego-depletion experiments in our lab and found we had trouble replicating the effect. However, we noted that many other labs had successfully replicated and extended Baumeister et al.’s effects. So, we resolved to conduct a meta-analysis of laboratory experiments testing the effect to provide point and variability estimates for the effect across studies and, importantly, whether or not there were moderators that determined the conditions under which the effect might, or might not, be observed.

Our meta-analysis included all studies using the received approach to testing the effect: a two-task paradigm in which participants completed an initial task which demanded (self-control condition), or did not demand (control condition), self-control, followed by a second separate task that required self-control. To the extent that performance on the second task was impaired under the self-control condition for the initial task relative to the control condition, the ego-depletion effect was confirmed. Importantly, we confined our analysis to published studies assuming, rather naively as turned out (‘I wish I had known … !’), that it provided a level of ‘quality control’ on the studies. The analysis yielded a medium-sized ego depletion effect (*d* = 0.62; Hagger, Wood, Stiff, & Chatzisarantis, 2010) across studies with substantive variability, and we also identified some candidate moderators such as task duration, intertask interim period, and task complexity, none of which, however, resulted in a null ego-depletion effect, but merely diminished or magnified its size. Nevertheless, we remained steadfast in our assumption that the difficulty we experienced in observing the ego-depletion effect in our lab was due to something we were doing ‘wrong’ despite our best efforts to follow the original paradigms.

However, not long after the publication of our meta-analysis on ego-depletion, a re-analysis and additional analysis conducted by Evan Carter and Mike McCullough appeared (Carter et al., 2015; Carter & McCullough, 2014). They reanalyzed our data applying stricter inclusion criteria and added unpublished data to their database. In addition, they applied a series of statistical correction techniques in their analyses that aimed to estimate the potential presence of ‘small study’ bias and correct for it. Specifically, the latter techniques are purported to detect the presence of disproportionately large effect sizes relative to their sample size in samples of studies in a meta-analysis, a potential indicator of publication bias – the tendency for authors or those involved in deciding what gets published to favor ‘positive’ research findings (Stanley & Doucouliagos, 2014). Carter et al.’s analyses revealed that the ego-depletion effect size was much smaller than reported in our meta-analysis, and, when applying the bias-correction techniques may so small as to be trivial and not distinguishable from zero! Although some concerns were expressed over the accuracy of the bias correction methods (Hagger & Chatzisarantis, 2014; Inzlicht, Gervais, & Berkman, 2015), the potential for bias was real and most disconcerting – had our focus on published studies inadvertently resulted in an inflated estimate of the ego-depletion effect? Was the ego-depletion effect yet another example of an effect that was difficult to replicate, similar to other high-profile cases that had been reported? These findings led to a lot of consternation among researchers in the field.

Around the same time, the Association of Psychological Science was launching their registered replication reports initiative, which was aimed at providing robust data for key effects in psychological science, particularly where concerns about replication had been flagged (Simons, Holcombe, & Spellman, 2014). In light of Carter et al.’s findings, the ego-depletion effect seemed like a prime candidate for replication. So, we set out to conduct a good-faith replication of the ego-depletion across multiple laboratories. We proposed the replication study to the APS, which was subsequently reviewed and accepted. The replication was a substantive undertaking involving procedures that were relatively innovative at the time including development of a standardized replication protocol using appropriate methods, peer review of the replication protocol, solicitation of support for the protocol from the proponents of original researchers – Baumeister and his colleagues, Chandra Sripada and Daniel Kessler, who supplied the paradigm (Sripada, Kessler, & Jonides, 2014), duly obliged – and recruitment of a requisite number of labs who pre-registered their predictions. We recruited 23 labs who carried out the ego-depletion experiment as specified in our protocol, and submitted their data for individual lab and meta-analysis. Results were very revealing – a very small, trivial effect size for the ego-depletion effect that was not discernible from zero, just as Carter et al.’s analysis had indicated (Hagger, Chatzisarantis, et al., 2016).

The findings found resonance with researchers in psychology and the popular scientific media alike. Media headlines included ‘Everything is Crumbling’ (Engber, 2016) and ‘The End of Ego-Depletion Theory’ (Neuroskeptic, 2016), and Michael Inzlicht, a prominent figure in self-control and social psychology, said: ‘I’m in a very dark place’ as he tried to come to terms with the replication failure and its implications (Inzlicht, 2016). Hyperbole aside, these statements captured the somber mood of the research community at the time. There was pushback and concerns were noted, some measured and some less so, but they too reflected the general concerns (Baumeister & Vohs, 2016; Lurquin & Miyake, 2017; Sripada, Kessler, & Jonides, 2016; Witte & Zenker, 2017). Our replication inspired further replications of the ego-depletion effect (e.g. Dang et al., 2020; Garrison, Finley, & Schmeichel, 2019; Lurquin et al., 2016), including one led by Baumeister’s colleagues Kathleen Vohs and Brandon Schmeichel (Vohs et al., 2021), and all revealed small effect sizes or effect sizes that were no different from the null. In many respects, this demonstrates the value of the replication and the ensuing debate – catalyzing additional research and providing more robust data to further build the evidence base and inform theory, no matter what the findings. Furthermore, these replications were not conducted by groups of ego-depletion sceptics, in fact many were proponents, ourselves included, but importantly they were united by a common interest in developing the best evidence regardless of the outcome.

My experience and direct involvement in this highly contentious series of events – and finding myself unexpectedly ‘caught in the middle’ of the ensuing debate – was highly educational and informative, and had an indelible impact on my approach to research. I developed a much deeper understanding of the potential for bias in research, particularly when it comes to published findings, and the potential for researchers’ questionable research practices, applied wittingly or unwittingly, to have a substantive impact on a research literature. The ego-depletion effects in the published literature were biased and served as a high-profile example of the ‘file drawer problem’ – null findings that do not fit with an established paradigm remaining unpublished, perhaps because researchers thought those effects were unlikely to be published or they had tried to publish them and failed. The replications had led me to alter my beliefs about the ego-depletion effect, demonstrating the value and power of replication as an agent for change (McDiarmid et al., 2021). It also meant that I became familiar with many of the principles being advocated by ‘open science’ groups at the time: the essentiality of sharing data and findings across labs, being upfront about research predictions and making them public them for all to see before conducting and analyzing observations, and the importance of robust data with sufficient sample sizes and statistical power based on reasonable a priori effect sizes. While I had been implicitly aware of many of these issues previously, the visceral first-hand experience with the ego-depletion research led to an epiphany of sorts that needed to be confronted, and I knew that I had to make drastic changes in my research mindset going forward.

### Meta-analysis and the fugitive literature

The experience with ego-depletion was an important ‘flashbulb moment’ that compelled me to make drastic changes to my research practices going forward. However, another catalyst for change, one which had perhaps a less transformational, but more organic, influence on my research practices, was my involvement in conducting meta-analyses. Since my doctoral studies, I have been conducting meta-analyses to synthesize research findings on the social psychological correlates of behavior derived from social cognition, motivational, and, more recently, integrated theories in numerous contexts and populations, and across multiple behaviors, particularly health-related behaviors (for a discussion see Hagger et al., 2020; Hagger, Chan, et al., 2016). My attraction to meta-analysis is its potential to provide overall estimates of effects and their variability based on available data that are adjusted for certain biases, such as sampling and measurement error (see Hagger, 2021). This enables a researcher to identify the ‘true’ variability in the effect across studies, and to search for possible moderator variables that may explain that variability. Another advantage is that they allow a researcher to test theoretical predictions, sometimes unique predictions, based on the available data. To this end, I have been involved in dozens of meta-analyses over the years, which have contributed to the evidence base of these theoretical correlates.

However, as the ego-depletion experience illustrated with extreme poignancy, meta-analyses are only as good as the included data. Our ego-depletion analysis was limited because it only included published studies, and highlighted the folly of not searching for and including unpublished data or ‘grey’ literature, which may not have found its way into the published literature due to some of the biases I outlined previously. Analysts cannot, therefore, rely on peer review as an adequate control on quality, given the inherent biases, and, as such, meta-analyses relying on published data are unlikely to provide reliable findings. Within meta-analysis, this is referred to as the ‘garbage in, garbage out’ phenomenon. So, inclusion of unpublished data is essential for integrity in meta-analyses, but getting access to these data presents considerable challenges to the analyst. Most meta-analysts or systematic reviewers will recognize the low incidence of comprehensive reporting of data across the research literature, and reporting is oftentimes woefully inadequate. Pursuit of this ‘fugitive literature’, as Rosenthal (1994) eloquently labeled it, requires effort and persistency. Nevertheless, even the most committed analyst will be fortunate to collect a substantive proportion of these unpublished data for a multitude of reasons – researchers move on from their positions, or even out of research positions altogether, so are difficult to contact, data files get destroyed, corrupted, or lost, particularly if the data are from studies conducted a long time ago, and some researchers are reluctant share their data. These issues are a meta-analyst’s bane, and mean that datasets used in most meta-analyses are incomplete at best, despite researchers’ best efforts. This introduces substantive bias to meta-analysis.

Recent shifts toward data sharing in psychology and other scientific disciplines promise much for the comprehensiveness and reliability of meta-analyses going forward. Catalyzed by the recommendations of open science proponents and organizations (e.g. Nosek et al., 2015), journals and research organizations (e.g. American Psychological Association, 2021; NIH, 2021b; NSF, 2021b) are encouraging, or even mandating, data sharing. Furthermore, increased trends in posting pre-prints of unpublished research online mean access to unpublished data and research reports is greater than ever. Of course, this does not resolve the issues of access to unpublished data from studies conducted prior to the introduction of these initiatives, but it illustrates a concerted effort by the scientific community to self-correct and improve reporting practices in research. Nevertheless, adequate reporting and archiving of data in psychology remains extremely limited, particularly in the health domain, and still has potential to introduce non-trivial bias in meta-analyses. My frustration in being unable to source data for the meta-analyses my collaborators and I conduct, has led me to recognize the need to ensure that my own practices are satisfactorily compliant. Together with the epiphany arising from the ego-depletion experience, my experiences with meta-analyses and the limitations caused by data unavailability has served as a learning moment that inevitably led me to the door of open science and the need make changes to my own research practices and to advocate similar changes in others.

## A commitment to open science and the open science ‘Mindset’

As a consequence of the dual experiences of the ego-depletion replication analysis and the pursuit of fugitive literature in meta-analyses, I made a conscious, concerted shift in my research approach toward open science, and to becoming an open science advocate. I resolved to, wherever possible, make all my research materials (e.g. questionnaires, intervention scripts, experimental protocols), data, and analysis methods (e.g. code for data analysis software, coding manuals) and output freely available, and to register my hypotheses and predictions in advance, for the research projects I was leading, and encourage my collaborators to do the same (for some recent illustrative examples in the literature, see Hagger & Hamilton, 2021; Heino, Vuorre, & Hankonen, 2018). To do so, I realized I would have to completely change my way of thinking on how I conducted my research. I found that the most optimal way to proceed was to commit to a basic assumption: everything I would do on a research project should be able to be seen by others. I therefore proceeded as if a group of hypothetical auditors were scrutinizing every step of the research projects my colleagues and I conducted. In short, I resolved to adopt an open science ‘mindset’. I viewed this as the ultimate commitment to openness and transparency with the goal of full compliance with the highest levels of these practices advocated by open science scholars and organizations, and recommended in published open science guidelines (e.g. Nosek et al., 2015; The FOSTER consortium, 2021a).

I also set about advocating for open science practices in the ways I could. An important way I could serve as an open science agent of change was to pro-actively make changes in the peer review and publishing practices at the scholarly journals for which I was chief editor. I subsequently worked with the co-editors, and editorial boards of these journals, *Stress and Health* and *Health Psychology Review*, to introduce open science practices, including encouraging or mandating open science materials, data, and pre-registration (Hagger, 2019; Probst & Hagger, 2015). The initiatives were supported by the publishers, who had been upping their open science game so as to become compliant with open science recommendations, which also coincided with decisions made by leading funders that publicly-funded research should be freely available to all rather than locked behind the paywalls of proprietary journals. My adoption of an open science ‘mindset’ and changing my own research practices was, in this regard, essential – editors advocating for open science have to ensure that their own house is in order so that they can lead by example. I continue to do this in my current co-Editorial role with *Social Science and Medicine*. Taken together, these changes were a culmination of my own experiences that highlighted the potential for bias in current research practices, and how changes in approach could minimize that bias, something I wish I had known earlier in my research career.

I also had the aspiration that my commitment to open science would make a contribution to future research practices through student education. I resolved to actively induct a new generation of researchers for whom adoption of an open science ‘mindset’ would not be conscious decision or represent a ‘new normal’, but rather the received way of conducting research. In this regard, I resolved to also serve to educate others on open science practices. My graduate students are introduced to open science from the outset of their graduate studies, so that they learn to adopt an open science ‘mindset’ from the very beginning of their doctoral studies, and open science practices such as data and materials sharing are integral components of their research methods. I have also become involved in workshops on open science at my own and other universities, scientific meetings, and as a service to the memberships of the learned societies to which I belong. In addition, my colleagues and I have published articles and commentaries on the subject of open science (e.g. Hagger, 2019; Kwasnicka et al., 2020), and have been involved with events that discuss optimal practices in open science at society meetings and with organizations that advocate for open science. These activities are aimed at raising awareness of the advantages of open science, and encouraging others to make changes to improve the transparency of their research practices and, in doing so, make a contribution to improving the quality and integrity of psychology research. Next, I outline the features of the open science mindset, and how researchers may go aboutassuming such a mindset going forward.

## Features of the open science ‘Mindset’

The open science ‘mindset’ is predicated on the assumption that every step of a research project, from inception to dissemination, will be publicly available and made accessible via widely used or available formats or platforms. Although the idea of giving the research community an ‘all areas pass’ to your research procedures may seem somewhat novel (and potentially daunting!), it is actually fully consistent with the practices advocated by open science proponents and the centuries-old purpose of research reports as a means to facilitate others in replicating the research exactly as specified. However, this purpose has been somewhat lost as researchers adopted a more closed mindset when reporting their research, most likely because of their prior training and some of the concerns outlined previously. Furthermore, many researchers see little reason for change, and hold beliefs that their research practices are already effective and fit-for-purpose. The onus is, therefore, on those that have embraced these practices to become open science advocates, and to highlight the advantages of developing an open science ‘mindset’, and to dispel misconceptions, through education and incentivization.

*Open science from inception to dissemination*: The first and most fundamental feature of the open science ‘mindset’ is the assumption all aspects of a research project will be open and freely accessible to any reader from the inception of the study, the stage when the research idea is first proposed, to completion, usually the dissemination or publication of the research findings. Adopting the mindset from the very first stages of a research project means that open science will be integral to the research rather than an afterthought, and ensures that the researcher has the opportunity to cover all relevant open science practices. This is particularly important for the submission of a study pre-registration or registered report (Jonas & Cesario, 2016; Nosek & Lakens, 2014), and securing clearance from participants and any other gatekeepers such as the IRB or institution ethical review committee to share anonymized data collected in the course of the project in future (Grant & Bouskill, 2019),^1^ two steps that cannot be done retrospectively. It also means the researcher will be able identify the necessary steps and procedures to ensure compliance with open science best practice, and designate tasks relating to open science to research personnel, such as documenting methods, and developing codebooks and interim materials.

*Consideration of broad access*: A second key feature of the open science mindset, is to develop a plan on how to share study data and the data analysis scripts that will be used as input for the analytic software or tools used (Schiermeier, 2018). Thinking ahead about what form the analysis and data will take, how best to share it, where to share it, when to share it, and to accumulate relevant permissions (e.g. participants, collaborators), and gain IRB/research ethics committee approval, would be essential components of such a plan. Researchers should consider making data available in commonly-available formats so that it is broadly accessible, which means using widely-available, non-proprietary software or applications (e.g. using file formats for digital text documents such as.pdf,.txt or.rtf files, and data spreadsheets formats such as.csv). Also important is the provision of codebooks or supporting information to accompany these materials and data files that provide sufficient information to enable researchers and other consumers of the open data and materials to interpret their content (e.g. definitions of the variables and the form they take in the data file, spelling out acronyms used in documents, or providing supplementary information on the data analysis tools used). This feature of the mindset requires investigators developing proposed research projects to consider in advance all data and analytic tools that other researchers and consumers would likely need to reproduce the study analyses exactly as specified in the original study.

*Selection of an open science forum*: A third feature of the mindset, allied to the first two, is the consideration of the forum, or fora, by which pre-registration and study materials (e.g. protocol, codebooks), data files, and analysis scripts will be made available, consistent with open science guidelines. This means identification of appropriate and reputable online services that enable permanent and reliable registration and archiving of studymaterials and data. Many registries and repositories are currently available that provide fit-for-purpose self-archiving services that comply with open science guidelines, and they are increasing in their useability and sophistication. In addition, they are often provided free-of-charge or for a relatively modest fee. In terms of registries, many exist, particularly in the fields of medicine and health sciences, and most are appropriate for those conducting research in the fields of health psychology and behavioral medicine. Some registries have an exclusive focus on interventions and trials (e.g. https://www.isrctn.com/), while others are more focused on research reviews (e.g. https://www.crd.york.ac.uk/prospero/). Research proposals submitted to these registries are usually subjected to an internal triage for appropriateness and completeness of content, but not for the quality of the research itself.

With respect to data repositories, most allow researchers to create ‘projects’ and organize their data and materials under an (usually) intuitive filing system, and are relatively generous in the size of the storage ‘space’ on offer. To be appropriate for open science, repositories need to provide a permanent and transparent record of the data and materials filed, such that they will not be deleted or lost unless the researcher chooses to do so. They should also keep a log of alterations and modifications, which is important for maintenance of transparency. Many repositories now provide free digital object identifiers (doi), a unique and permanent link to the data and materials, that can be cited in research reports, journal articles, and other forms of research dissemination. Good summaries of available repositories and their features have been published (e.g. https://www.nature.com/sdata/policies/repositories; http://oad.simmons.edu/oadwiki/Data_repositories). An example of a widely use repository that is likely to suit the needs of most researchers in health psychology and behavioral medicine is the Open Science Framework (OSF; https://osf.io/). The OSF provides pre-registration and archiving services all ‘under one roof’.

Another option is to consider archiving materials and data with the journal or publication to which the final report is submitted. Publishers of scientific journals are becoming increasingly aware of the need to provide these services, and some even mandate it, so the research needs to explore this with the journal editor or publisher in advance if this is the option they plan to take. The drawback is that the materials will not be made available except to the scholars involved in reviewing the article until the research published, so researchers often opt to archive their materials and data using an online repository.

A further option is to consider a registered report, a type of journal article that involves a two-stage review process (Nosek & Lakens, 2014). In the first stage, the research protocol, essentially a comprehensive form of a research pre-registration, is submitted to the journal and subjected to peer review prior to the conduct of the research and data collection. If the submission passes peer review, it is provided with ‘in principle’ acceptance for publication regardless of the findings pending subsequent re-review at the second stage to ensure the research was conducted as proposed. Sharing materials and data is expected for authors opting to submit a registered report as the goal is to maximize transparency and promote compliance with a confirmatory, hypothesis-testing approach. The onus is on investigators to explore the pre-registration and archiving options most suitable for their project, and, most importantly, to keep this in mind from the inception of their projects all the way through to final dissemination.

*Open and transparent dissemination*: An open science mindset also means paying close consideration to the most appropriate means to make final reports of research projects freely available. A traditional approach to research dissemination is through publication of articles in peer-reviewed journals owned by publishing houses that charge scholarly institutions, professional organizations, and individuals to access to their journal catalog. The consequence of this practice means that much published research remains behind the ‘paywalls’ of those publishing houses severely limiting access to research. This restrictive practice not only limits access to the general public, but also accentuates the disparities in access to research evidence between high and low income institutions, nations, and individuals (Grahe, Cuccolo, Leighton, & Cramblet Alvarez, 2020). These access issues have recently come into sharp focus through lobbies for research supported by taxpayer-funded grants to be made publicly available. This pressure has resulted in the formation of groups such as cOAlition ‘S’, an international consortium of research funders who, through plan ‘S’ (‘S’ for ‘shock’), have instigated sweeping changes to research dissemination by working with publishers and researchers to make all publicly-funded research open access (cOAlition, 2021). More broadly, such initiatives have coincided with the rapid rise of open access journals in which all articles are made openly available, usually under a Creative Commons Attribution license (CC BY) license, or hybrid journals which offer the option of open access, both of which shift the cost of publication to the author or their funder. This is known as a ‘gold’ open access publishing model, in which submitted research is made openly available on acceptance to the journal after a peer review process. The drawback is that fees are often high, and out of reach of many individual researchers, or place an undue burden on researchers and funders from low – or middle-income countries, with publishers’ pledge of open access fee waivers often confined to a small number of countries. ‘Green’ open access is an alternative in which the accepted manuscript version of a research article published in a subscription journal is self-archived by the author, often after an embargo period. There has also been a rise of journals that offer ‘platinum’ or ‘diamond’ open access, in which research articles are published fully open access with no publication fee. Sherpa/Romeo (https://v2.sherpa.ac.uk/romeo/) hosts a searchable database of journals allowing a researcher to identify the type of open access options publishers offer, including the embargo on a ‘green’ open access.

Researchers with an open science mindset will also consider ‘pre-printing’ their unpublished manuscripts prior to publication, a well-established practice in some areas of science, and on the rise in many others (see Coudert, 2020). Pre-printing entails authors depositing reports of the findings of research projects on an online server designated for that purpose on their completion and prior to formal publication. Many free-of-charge online archiving servers are available, such as PsyArxiv for psychology-oriented pre-prints, which provide a permanent record of the archived manuscript and offer the opportunity to mint a unique doi free of charge. Archiving a pre-print has numerous advantages, including soliciting early feedback from the research community, allowing the research to attract citations and for the research team to cite their own research in subsequent work, and producing a log of when the research was completed and subsequent revisions (Sarabipour et al., 2019). It also allows for self-archiving of the final manuscript version, consistent with a ‘green open’ access sharing policy.

Researchers adopting an open science mindset have an obligation to make their research open access to the research community and general public, and should consider the most appropriate option from those outlined above to do so. Key to this feature of the mindset is to draft an archiving and dissemination plan, and identify the outlet(s), from the outset of the research, rather than making such decisions post hoc. Of course, final publication of research is reliant on the write up of the research passing the peer review process, so it would be prudent for researchers’ not to confine their planned publication to one journal or outlet, but to identify a candidate list of journals that fit well with their planned open access option and dissemination strategy. This, of course, may be obviated by a decision to select a journal with a registered report article option (see feature 3, above), in which case the journal usually has an obligation to publish the research open access because this article type incorporates transparency and pre-registration as standard.

*Open science advocacy*: Adopting an open science ‘mindset’ means making a commitment to open science, and those adopting this mindset have an obligation to ensure that, at the very minimum, those involved in their program of research are literate in, and agree to participate in, the planned open science practices of a research project. In my own program of research, having this ‘mindset’ means I am compelled to highlight the benefits and advantages that adopting open science approach confers, and make my commitment to open science clear. I do this from the very beginning of a collaborative research project and make it a central component of the research partnership. The strategies that I employ to do so is through communication and modeling. I discuss open science issues with the research team from its outset and outline my commitment to them, and give examples of my previous projects that have incorporated open science practices. This is to ensure that all those working on projects are clear about my goals for transparency and the planned means to ensure they are realized.

Given that open science is yet to become standard practice among psychology researchers, I believe that adopting an open science mindset also means to become an open science advocate more broadly (see for example Armeni et al., 2021; The FOSTER consortium, 2021b). Researchers can do many things to raise awareness of and advocate for open science practice. For example, many researchers working in academic contexts are involved in the delivery of teaching and training to undergraduate and graduate researchers. This presents a unique opportunity to inform and educate the next generation of researchers in open science practices, and to foster an open science ‘mindset’ in those likely to be integral to shaping research norms in future. It is through pro-active education in, and the modeling of, open science practices by researchers for their students in such contexts that open science will shift from being the ‘new normal’ to the established, received means to conduct and disseminate research going forward (see also Jekel et al., 2020; Ortha, Pontika, & Ball, 2016; Steinhardt, 2020; Strand & Brown, 2019). Research can also advocate for open science by highlighting open science practices when serving as a peer reviewer of manuscripts submitted for publication (Morey et al., 2016). Importantly, my focus, and those of many open science advocates, has always been to incentivize and encourage the adoption of these practices, highlighting their benefits to the researcher and the research community at large as opposed to applying pressure to adopt them or imposing diktats. That said, I recognize that rule changes and regulations are necessary to ensure that these practices become routine and highlight the importance that organizations and publishers assign to these practices.

### Caveats and issues

Researchers have voiced concerns about the adoption of some open science practices, and these should be acknowledged. Some concerns are the result of misconceptions (see Open Science Community Groningen, 2019), and the open science advocate has a duty to provide evidence based responses in a supportive, non-confrontational manner, while highlighting the benefits of open science. In addition, concerns that have not arisen through misconceptions can be mitigated through open science practices that effectively manage or obviate those concerns. Concerns that have been cited by researchers include beliefs that pre-registering or pre-printing their research, and sharing data and materials, will lead other researchers to ‘steal’ their ideas or ‘scoop’ them to their research finding, and beliefs that pre-registration of predictions and analysis plans are unnecessarily restrictive and serves to stymie creativity and straightjacket the researcher from performing exploratory analyses (Olson, 2021; Open Science Community Groningen, 2019). Others fail to see the advantages of open science practices, or feel that it adds substantially to the workload of a research community that is already under pressure for time and resources, with little payoff. Therefore, the open science ‘mindset’ may, to some, seem to pose more disadvantages than advantages, and goes against the grain of established research practices. Positive advocacy and education may help in changing these views by highlighting false beliefs and misconceptions on open science in a non-confrontational, supportive way, along with highlighting that the advantages of adopting these practices outweigh the disadvantages and that the outcomes are well worth the effort involved.

Effective communication of open science practices can contribute to dispelling false beliefs, demystifying concerns, and challenging misinformation about open science. For example, pre-registration does not in any way rule out additional exploratory analyses conducted alongside the pre-registered analyses, and enables the researcher to be transparent on which analyses were pre-registered and which were exploratory (Nosek et al., 2019; Olson, 2021; Open Science Community Groningen, 2019). It should also be highlighted that pre-registration of research protocols and hypotheses, and pre-printing manuscripts reporting research, will serve to mitigate ‘scooping’ by providing a means to attribute research ideas and findings to the authors that is time-stamped, linked to the author, and uniquely traceable and referable (e.g. through a doi or unique link). Similarly, advocacy can highlight the distinct advantages that open science can offer such as the greater likelihood that pre-printed research will be included in reviews and analyses, and will receive reads and citations (Fraser, Momeni, Mayr, & Peters, 2020). Taken together, broad adoption of an open science ‘mindset’ necessitates effective advocacy work, as I highlight in the final feature of the open science mindset above, to reassure scientists of the benefits of open science, and challenge or mitigate the concerns and misconceptions they may hold.

Acknowledging the need to adopt open science practices at all stages of research also implies that such practices should apply to all types of research. Much of what has been written about open science has tended to have a heavy emphasis on quantitative research, largely because many of the questionable research practices identified have been linked to this type of research. This emphasis is apparent in the current article as my reflective experiences are drawn from quantitative research. However, there is increasing recognition that open science practices should equally apply to qualitative research. In fact, some of the fundamental principles of qualitative research lend themselves directly to open science (Humphreys, Lewis, Sender, & Won, 2021), such as the principle of reflexivity, that is, researchers acknowledging their fundamental role in the research and data generation process (Guzzardo et al., 2021). This highlights need for maximum transparency when it comes to making such acknowledgements, and the need to provide sufficient supporting evidence.

Open science advocates (e.g. Renbarger, 2021) have therefore highlighted the need for researchers adopting qualitative methods to make their raw (e.g. interview transcripts, audio – and video-recordings) and processed (e.g. theme extraction, notes from iterative readings, content analysis) data open, provided adequate permission or consent has been given, and to provide clear details of their analytic approach and methods (e.g. philosophical approach, notes during theme coding, protocol for handling disagreements between coders) when reporting research (but see also Chauvette, Schick-Makaroff, & Molzahn, 2019 for an alternative perspective). Pre-registration of qualitative research has also been advocated. Open science proponents have also pushed back on the notion that pre-registration of qualitative research is contraindicated because qualitative methods are inherently interpretive and exploratory (‘postdiction’ rather than ‘prediction’; Haven & Van Grootel, 2019). However, this represents a mis-characterization of qualitative methods (see Hagger & Chatzisarantis, 2011), and pre-registration of qualitative studies can highlight aspects of the methods and analyses that are predictive and those that are likely to be emergent or interpretive, similar to confirmatory and exploratory analyses in quantitative research. Readers interested in the application of open science practices in qualitative research should consult the available open science reommendations for this type of research for further guidance (e.g. Humphreys et al., 2021; Renbarger, 2021).

## Conclusion

Open science is here to stay – it is becoming increasingly important in the context of the need for replication, transparency, and trustworthiness in psychology research, particularly in light of high-profile replication ‘failures’, and evidence of potential bias in effects. Many researchers, publishers, funding organizations, and professional organizations that represent researchers and organize scientific meetings are putting means in place to ensure research findings and all aspects of the research processes are performed and disseminated, wherever possible, in accordance with open science guidelines. Adoption of an open science ‘mindset’, in which researchers adopt a ‘farm to fork’ approach where open science is a central feature of research projects from inception to dissemination, is a highly effective means to ‘leave no stone unturned’ when it comes to adopting open science practices. An open science mindset, therefore, represents a comprehensive, integrated approach to open science, ensuring that no aspect or step is intentionally missed or omitted along the way, and is aimed at realizing the goal of maximum transparency. Given my experiences with meta-analyses, including my experienced frustrations arising from biases in psychological research and the challenges in tracking down data that is not published in the original reports of included studies or unpublished data, I wish I had known earlier about the advantages open science. However, I have no regrets: these experiences led me serendipitously to the door of open science, and highlighted the advantages of adopting an open science mindset in my own research and those of my colleagues and collaborators, and compelled me to become an advocate for open science practices going forward.

## Data Availability

There are no data associated with this article.

## Note

1 It is important to recognize that some data cannot be shared openly due to confidentiality concerns (e.g., some data can lead to the indentification of individuals even if deanonymized) or because certain types of data are particularly sensitive. These limitations are acknowledged and accepted by those advocating open science. Researchers working with data to which such restrictions apply are, therefore, encouraged to flag the issues and provide a clear rationale for not sharing. However, such restrictions do not, for example, preclude the possibility of sharing other aspects of the research that will promote transparency, such as sharing the study materials used to collect the data and the data analysis scripts and output.
